# Genes, Pathways, and Mechanisms Involved in the Virulence of Mucorales

**DOI:** 10.3390/genes11030317

**Published:** 2020-03-16

**Authors:** Carlos Lax, Carlos Pérez-Arques, María Isabel Navarro-Mendoza, José Tomás Cánovas-Márquez, Ghizlane Tahiri, José Antonio Pérez-Ruiz, Macario Osorio-Concepción, Laura Murcia-Flores, Eusebio Navarro, Victoriano Garre, Francisco Esteban Nicolás

**Affiliations:** Departamento de Genética y Microbiología, Facultad de Biología, Universidad de Murcia, 30100 Murcia, Spain; carlos.lax@um.es (C.L.); carlos.perez6@um.es (C.P.-A.); mariaisabel.navarro3@um.es (M.I.N.-M.); josetomas.canovas@um.es (J.T.C.-M.); ghizlane.tahiri@um.es (G.T.); joseantonio.perez6@um.es (J.A.P.-R.); macario.osorio@um.es (M.O.-C.); lauramur@um.es (L.M.-F.); vgarre@um.es (V.G.)

**Keywords:** Mucorales, mucormycosis, *Mucor*, *Rhizopus*, iron, dimorphism, calcineurin, PKA, RNAi, antifungal resistance, epimutant, azole, azole resistance, lanolase, host-pathogen interactions, CotH

## Abstract

The order Mucorales is a group of ancient fungi with limited tools for gene manipulation. The main consequence of this manipulation unwillingness is the limited knowledge about its biology compared to other fungal groups. However, the emerging of mucormycosis, a fungal infection caused by Mucorales, is attracting the medical spotlight in recent years because the treatments available are not efficient in reducing the high mortality associated with this disease. The result of this renewed interest in Mucorales and mucormycosis is an extraordinarily productive effort to unveil their secrets during the last decade. In this review, we describe the most compelling advances related to the genetic study of virulence factors, pathways, and molecular mechanisms developed in these years. The use of a few genetic study models has allowed the characterization of virulence factors in Mucorales that were previously described in other pathogens, such as the uptake iron systems, the mechanisms of dimorphism, and azole resistances. More importantly, recent studies are identifying new genes and mechanisms controlling the pathogenic potential of Mucorales and their interactions with the host, offering new alternatives to develop specific strategies against mucormycosis.

## 1. Introduction

The evolution of microorganisms represents a continuous origin of emerging human pathogens, new strains of bacteria and fungi that become resistant to our current antibiotic and antifungal compounds. Among fungi, the order Mucorales is a source of antifungal resistance species identified as the causal agents for the lethal and emerging disease known as mucormycosis [1]. Mucormycosis is a fungal infection that represents the third most common angio-invasive fungal infection after candidiasis and aspergillosis [2]. Among Mucorales, species of the genera *Rhizopus*, *Mucor*, and *Lichtheimia* are the most frequent causal agents for this fungal infection [3]. Significant increases in mucormycosis cases, mortality rates of 90% in disseminated infections, and the lack of effective antifungal treatments have raised the alarm on this emerging disease. In the past, it was considered a rare infection that was limited to immunocompromised patients suffering AIDS, diabetes, organ transplant or other conditions associated with immunosuppression [4]. However, the current improvement in the diagnosis techniques has revealed an alarming number of cases of mucormycosis in immunocompetent/otherwise healthy individuals [5].

Mucorales are a neglected phylogenetic group compared to others such as *Ascomycetes* and *Basidiomycetes.* The limited knowledge about the genetics of Mucorales is a consequence of the restricted tools for gene manipulation, as most of them cannot be transformed. However, DNA can be introduced in *Mucor circinelloides*, *Rhizopus delemar*, and *Rhizopus oryzae* [6,7]. These genetic models and the alarm raised for the emerging cases of mucormycosis are attracting the interest of the scientific community. Thus, the last decade has produced several studies related to genes, pathways, and mechanisms showing a direct connection with virulence in Mucorales [8,9]. One of the most studied mechanisms has been the process of gene silencing or RNA interfering (RNAi) in *M. circinelloides* [10]. After the dissection of the gene silencing machinery, the knowledge of this mechanism allowed the unveiling of a new and particular type of antifungal resistance mediated by temporal epigenetic changes [11]. In addition, the applied use of gene silencing enabled the development of functional genomics techniques, which have been used for the identification of several new virulence factors [12]. Along with silencing, gene disruption driven by homologous recombination has also allowed the study of the particular role in *M. circinelloides* of virulence factors identified in other fungi, such as the role of a high-affinity iron uptake mechanism, the protein family of CotH, and the calcineurin pathway. Moreover, the implementation of the new omics technologies has produced a long list of candidate genes not previously related to virulence, becoming promising targets for the development of new treatments against mucormycosis. Finally, the diversity of molecular and cell methodologies allowed the study of the genetic response during host-pathogens interactions, revealing the fundamental role of several regulatory genes [13]. Here, we summarize the results of these studies, showing the main advances in the knowledge of Mucorales fungi, the disease that they produce, and the perspectives for the development of effective future treatments.

## 2. Host Iron Uptake is A Key Element in The Pathogenicity of Mucorales

Iron is a key element needed for the survival of fungi. In tissue fluids such as plasma, free iron is not available because it is chelated by specific host mechanisms, being the most relevant ferritin and lactoferrin [14]. When these systems fail, the high free iron levels in the blood raise the susceptibility to mucormycosis as in patients with acidosis, including hyperglycemia and diabetic ketoacidosis (DKA). The abnormal low pH in the blood destabilizes the plasma chelators, facilitating the dissociation of iron from the proteins, and elevating the concentration of free iron in serum [15]. Fungal pathogens possess sophisticated mechanisms to compete and steal iron from the host. In this sense, Mucorales show two different strategies for iron acquisition: a high-affinity iron uptake system and the production of siderophores [16,17]. 

The high-affinity iron uptake mechanism has been widely studied in Mucorales. This system is composed of three proteins: an iron reductase (Fre), a ferroxidase (Fet3), and an iron permease (Ftr1). In this triad, the permease and the ferroxidase are the key proteins for the correct functioning of the system. Iron levels in the medium strictly regulate these three genes [17]. Thus, low availability of iron triggers the expression of the high-affinity iron uptake mechanism in *Rhizopus delemar* [18], *M. circinelloides* [17], and *Lichtheimia corymbifera* [19]. In fact, the expression of the ferroxidase genes is controlled by low iron concentration when the medium is treated both with synthetic iron chelators (like 1,10-phenanthroline) and with fetal bovine serum. The ferroxidase genes are also controlled during in vivo infection, being overexpressed in the lung of mice confronting *M. circinelloides* invasion [17]. The depletion of this high-affinity iron uptake mechanism results in decreased virulence of Mucorales, indicating that it is key virulence factor of mucormycosis. For instance, the deletion of the *ftr1* gene in *R. delemar* provoked defects in growth under low iron availability and reduced virulence in mice that suffered DKA [18]. Furthermore, iron starvation in the *R. delemar* strain deficient in Ftr1 induces a metacaspase dependent apoptotic process, confirming its critical role [20]. These results corroborate the relevance of the permease protein in the iron uptake during the infection, needed for the fungal survival under low iron concentrations.

In *M. circinelloides*, three ferroxidases genes have been characterized, named *fet3a*, *fet3b*, and *fet3c* [17]. Single and double deletion mutants in these genes demonstrated that they are crucial for the iron uptake when it is scarce in the medium and during the infection of a mouse model. Among the three gene copies, *fet3c* is the most relevant for the infection; however, the double mutant strains revealed a partial redundancy of the other two ferroxidases in the absence of Fet3c. Surprisingly, the ferroxidases genes in *M. circinelloides* not only carry specialized functions in iron uptake, but they are also subfunctionalized in dimorphism. The transition from yeast to mycelium is key in the virulence of *M. circinelloides* (discussed in the next section). Thus, dimorphism differentially regulates the ferroxidases genes in the two dimorphic states. The gene fet3a is overexpressed during yeast state, while fet3b and fet3c are involved in the iron uptake during hyphal growth [17]. These results suggested a functional specialization of the high-affinity iron uptake mechanism in dimorphism, linking two crucial virulence determinants of mucormycosis.

The use of siderophores is another crucial evolutionary acquisition to compete with the host for the iron available in the environment. Siderophores are small molecules that sequester iron with high affinity. Fungi synthesize different siderophores and can misappropriate siderophores synthesized by other microorganisms as xenosiderophores [21]. Mucorales can use both strategies to chelate and obtain iron from the medium. Rhizoferrin is the most characterized endogenous siderophore produced by several species of Mucorales [22]. However, the role of this polycarboxylate siderophore as a virulence factor remains unknown [16]. In contrast, the use of xenosiderophores by *Rhizopus* spp. has been widely linked with pathogenesis. Deferoxamine is the most frequent xenosiderophore involved in mucormycosis cases. It is a clinical siderophore used in dialysis treatments that can be utilized by *Rhizopus* spp. to chelate iron from the transferrin, forming the complex ferrioxamine (deferoxamine and iron). Ferrioxamine is then bound to the cell wall by Fob1 and Fob2 proteins, where the iron is transported inside the cell by the high-affinity iron complex [23]. Both receptors are necessary for the use of deferoxamine as a xenosiderophore, and their depletion provokes a reduced virulence in mice treated with deferoxamine [23].

Altogether, the iron uptake mechanisms in Mucorales are crucial for their survival inside the host and the dissemination of the infection. In consequence, the components of both systems are considered virulence factors that could be targeted by antifungal drugs. The substitution of deferoxamine by other iron chelators, such as deferasirox, is a recent example of the clinical efforts to prevent and treat mucormycosis. In vitro and in vivo studies demonstrated that deferasirox provoke iron starvation in Mucorales, suggesting that the administration of this chelator as an adjuvant could reduce the fungal dissemination [24]. However, the use of chelators combined with antifungals remains uncertain due to recent studies showing the adverse effects of this combined therapy [25]. Other worthy attempts to target the iron uptake mechanism in Mucorales include the passive immunization with anti-Ftr1p immune serum in mouse models [18] and the addition of *Pseudomona aeruginosa* siderophores to *Rhizopus microsporus* spores in the zebrafish larval model [26].

## 3. Dimorphism Controls the Pathogenic Potential of *Mucor circinelloides*

Dimorphism is the capacity of some fungi to alternate between yeast and mycelium during their vegetative growth. Several species of Mucorales show this type of growth shifting mediated by the environmental conditions [27]. In *Mucor* spp., anaerobiosis and the presence of a fermentable hexose induce yeast growth, whereas aerobiosis and nutrient limitation conditions lead the hyphal growth [28]. However, not all Mucor dimorphic species respond in the same way to environmental factors. Thus, *Mucor rouxii* needs both anaerobic conditions and the presence of a hexose to grow as a yeast, whereas *Mucor genevensis* can form yeasts under aerobic conditions in the presence of a high concentration of hexose [28]. There are also chemical compounds inhibiting the mitochondrial function that can induce the yeast form, even in aerobic conditions, such as inhibitors of the electron transport chain, oxidative phosphorylation, and inhibitors of the synthesis of mitochondrial proteins [27,29]. These observations suggest an active connection between aerobic respiration and the morphology of *Mucor* spp. Besides aerobic respiration, several studies also linked dimorphism with the metabolism of nitrogen in some *Mucor* spp. Thus, in *M. rouxii* and *Mucor bacilliformis*, the activity of the enzyme ornithine decarboxylase (ODC), which catalyzes the formation of putrescine from ornithine, increases during the yeast-hyphal transition and competitive ODC inhibitors, such as diamine butanone, block the yeast-mycelium transition in these species [30].

**Figure 1:**
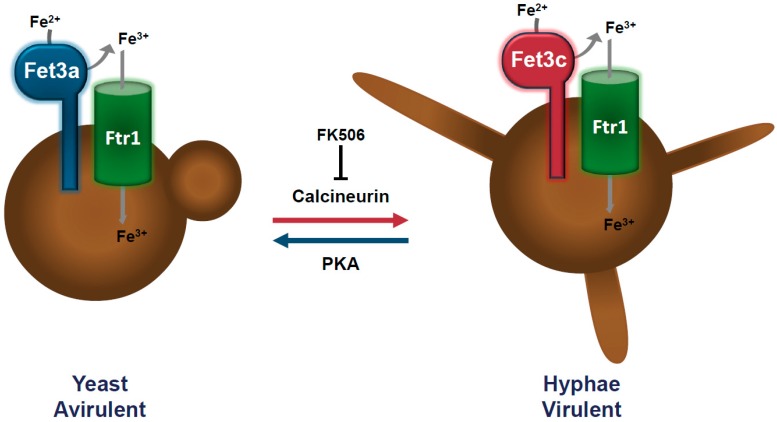
The interplay of calcineurin, protein kinase A (PKA), and iron uptake systems in dimorphism and virulence of *Mucor circinelloides.*

Recent studies found a link between dimorphism and virulence, becoming a new and promising target to develop compounds against mucormycosis. *M. circinelloides* is one of the *Mucor* spp. displaying dimorphism, growing as multi-budded yeasts in anaerobic conditions, and as hyphae forming a mycelium in aerobic conditions [27]. It is an exceptional fungus among Mucorales due to its genetic tractability and the wide range of genetic tools available for molecular studies [31,32,33,34]. Analysis of the calcineurin pathway in *M. circinelloides* revealed that it regulates the yeast-mycelium transition and virulence (Figure 1) [29,35]. Calcineurin is a serine-threonine phosphatase dependent on Ca^2+^/calmodulin formed by a complex of two subunits, the catalytic subunit A with phosphatase activity and the regulatory subunit B, which binds the calmodulin bound to calcium and activates the enzymatic complex. As in other dimorphic fungi, calcineurin performs its function by dephosphorylation of transcription factors that are transported to the nucleus, resulting in the expression of target genes [36]. *M. circinelloides* has one regulatory B subunit (CnbR) and three catalytic A subunits (CnaA, CnaB and CnaC). Disruption of the CnbR gene or the addition of calcineurin inhibitors results in mutants locked in the yeast phase and less virulent, showing that the dimorphic transition contributes to the virulence of this fungus [29,35]. Mutants in the *cnaA* gene remain in the hyphal growth state, but they have abnormal polarity, hypersensitivity to calcineurin inhibitors, cell wall defects and larger spores. These mutants are more virulent than the wild-type strain, as the larger size of the spores is also related to higher virulence in *M. circinelloides* [37]. 

Besides the calcineurin pathway, other pathways control dimorphism in *M. circinelloides*. It is the case of cyclic AMP (cAMP) and its target enzyme cAMP-dependent protein kinase A (PKA) [27]. PKA is a tetrameric holoenzyme with two regulatory (PKAR) and two catalytic (PKAC) subunits [38]. Three out of the four *pkaR* genes of *M. circinelloides* are implicated in the dimorphic transition. Over-expression of *pkaR1* promotes mycelial growth, whereas its deletion results in defects in the yeast-mycelium transition (Figure 1) [38]. PKAR4 is essential for the viability of this fungus, but the heterokaryotic mutant in *pkaR4* shows a defect in germ tube emergence when it changes from anaerobic to aerobic conditions, suggesting that PKAR4 is also involved in dimorphism. The absence of *pkaR2* promotes the transition from yeast to hyphae, indicating that PKR2 works as a repressor of this cellular process [38]. Interestingly, calcineurin negatively regulates PKA, suggesting a relationship between these two regulatory pathways [29]. Other proteins might also be involved in the regulation of *M. circinelloides* dimorphism, such as heterotrimeric G proteins and the ADP-ribosylation factors (Arfs), which are differentially expressed during the dimorphism process of this fungus [39,40]. 

## 4. RNAi in Mucorales and Its Role in Their Antifungal Drug Resistance

The elevated mortality rate of mucormycosis is due in part to the high antifungal drug resistance observed in the causative agents [41]. This unusual antifungal resistance demands new studies to understand the molecular basis of the mechanisms involved, with the final goal of improving treatments. *M. circinelloides* can develop drug resistance to antifungal agents through an RNAi-based pathway [11]. RNAi is a highly conserved mechanism among eukaryotes based on the production of small non-coding RNAs (sRNAs). Hybridization between those sRNAs and target transcripts leads to mRNA degradation or translation inhibition. The protein core involved in RNAi are RNA-dependent RNA polymerases (RdRPs) that synthesize the complementary strand of the transcripts to produce dsRNA [42]. The dsRNAs are processed by an RNase III endonuclease Dicer (Dcl1 or Dcl2) into small dsRNAs of 21-25 nt in size [43], which are loaded into the RNA-induced silencing complex (RISC), where the main protein is the Argonaute. This enzyme degrades one strand from the dsRNA and uses the other to recognize the target mRNA [44]. 

Modification of the central core by adding or removing components generate different types of pathways. For instance, *M. circinelloides* has three different RNAi pathways: the siRNAs-esRNAs pathway, the non-canonical *rdrp*-dependent *dicer*-independent regulatory pathway and the epimutational mechanism [11,45,46,47,48]. The siRNAs-esRNAs and the epimutational pathways are canonical in the sense that they require the participation of at least one Dicer and one Argonaute protein for the production of sRNAs [11]. The siRNAs-esRNAs pathway produces siRNAs as a defense against invasive nucleic acids. In contrast, esRNAs are produced to regulate physiological processes such as growth, sporulation, and autolysis [43,44,45]. The epimutational pathway shares most of the core proteins involved in the canonical pathway and is responsible for the phenotypic plasticity of the fungus under stressful conditions like the presence of antifungal drugs [11]. Thus, the epimutant strains specifically produce sRNAs against the target gene of the drug, suppressing its expression temporarily through mRNA degradation. After several passages in a free-drug media, epimutants re-expressed the gene target and became sensible again to the antifungal drug [11]. Recent studies showed that this human pathogen develops resistance to multiple antifungal drugs using this pathway [41,49]. In addition to core RNAi proteins, two other proteins act like activators of this epigenetic mechanism. These are an exonuclease similar to the quelling induced protein (QIP) and a Sad-3-like helicase (RnhA). The exonuclease QIP also participates in the siRNA-esRNAs pathway, and the helicase RnhA acts positively in the non-canonical mechanism. Loss-of-function mutations in the corresponding genes *qip* and *rnhA* block the production of epimutants [47]. 

**Figure 2:**
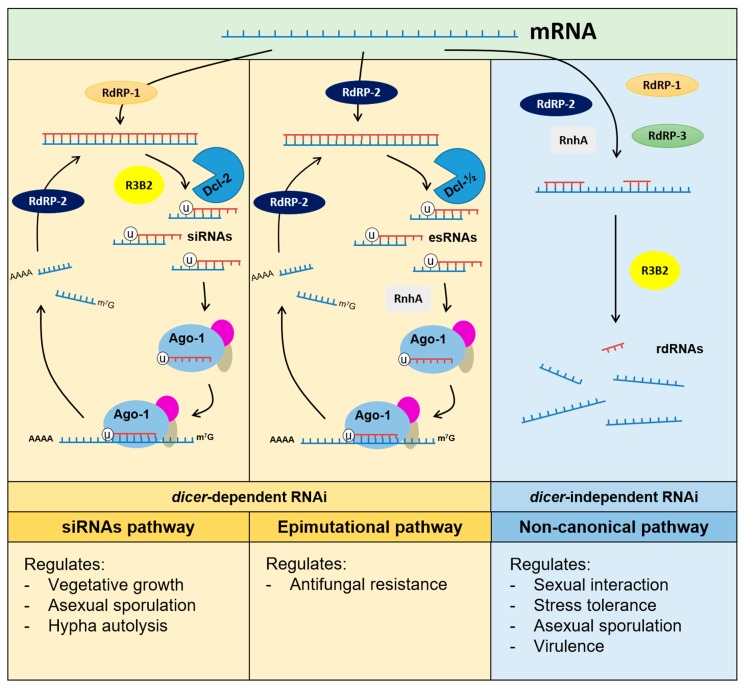
Diversity of RNA interfering (RNAi)-based pathways in *Mucor circinelloides*. RdRPs: RNA-dependent RNA polymerases; Dcl: Dicer enzymes; Ago: argonaute proteins; RnhA: Sad-3-like helicase; R3B2: RNaseIII domain (r3) and two dsRNA binding domain (b2); siRNAs: small interfering RNAs; esRNAs: endogenous small RNAs; rdRNAs rdrp-dependent dicer-independent RNAs.

On the other hand, the non-canonical pathway is particularly interesting because Dicer is not involved and the RNAse activity relies on a new protein called R3B2, which is specific to basal fungi [48]. The RNase R3B2 degrades transcripts, producing sRNAs with a non-discrete size that show a strong bias for cuts two nucleotides downstream of any uracil in the mRNA. This pathway controls the expression of diverse endogenous genes involved in metabolism and multiple cellular processes, such as mRNA maturation and heme biosynthesis. Besides its integral role in the non-canonical pathway, the R3B2 protein also participates in the Dicer-dependent mechanism, establishing a link between the two RNAi mechanisms [48]. Interestingly, the non-canonical RNA degradation pathway inhibits the epimutational mechanism. Thus, mutants defective in R3B2 promote the activity epimutational pathway and enhance the production of drug-resistant strains, suggesting that the two mechanisms may compete for the same mRNAs. However, while the epimutational pathway operates in response to challenging environments, the non-canonical mechanism functions during optimal growth [47]. The multiple interactions between the three RNAi pathways described in *M. circinelloides* reveal the complexity of these regulatory mechanisms (Figure 2). Further molecular studies of these connections will help to understand their participation in pathogenesis and virulence of Mucorales.

The ability of *M. circinelloides* to develop transient resistance to multiple antifungal drugs using the RNAi mechanism represents a new opportunity to developed treatments against mucormycosis. For example, the development of antifungal agents targeting the specific components of the epimutational pathway could represent a promising therapy to face the drug resistance of these fungi. QIP and RnhA constitute new potential targets to block the epimutational pathway and reduce drug resistance, as they are specific targets in fungi with low homology in mammal hosts. Similarly, the RNase R3B2 is conserved only among basal fungi, although its role in virulence is still uncertain.

## 5. Azole Resistance in Mucorales

Azoles are heterocyclic compounds broadly used for the treatment of fungal infections. Azole drugs containing three nitrogen atoms on its cycled structure are classified as triazoles. This group includes frequently used antifungal like fluconazole, itraconazole, voriconazole, ravuconazol, or posaconazole. The action mechanism of triazoles consists in the inhibition of the cytochrome P450 (CYP)-dependent lanosterol 14α-demethylase, an enzyme that catalyzes the transformation of lanosterol into 4,4-dimethylcholesta-8,14,24-trienol in the pathway for the production of ergosterol [50]. A decrease of the ergosterol levels in the membrane combined with the accumulation of toxic methylsterols is the cause of the fungistatic or fungicidal activity of these triazoles [51,52]. 

Triazole resistance has been reported and characterized over the years. Nowadays, it is a global health concern due to the increasing difficulties in the treatment of fungal infections, besides the high mortality and morbidity rates associated, which are a more crucial problem in the cases of immunocompromised patients [4]. Resistance to triazole drugs in Mucorales is also a serious concern, with the aggravating circumstance of being less studied than in other fungi. Nevertheless, in vitro and in vivo susceptibility tests have been performed for some of the most commonly used triazole drugs in a representative group of pathogenic Mucorales such as *Lichtheimia* spp., *Rhizopus* spp., and *Mucor* spp. The results show an intrinsic resistance to fluconazole and voriconazole, both lacking significant in vitro activity against Mucorales [1,40,53]. However, posaconazole shows in vitro activity with MIC_50_ ranging between 0.125 and 1 µg in *Lichtheimia* spp., 0.25 and 2 μg/mL in *Rhizopus* spp., and 0.5 and 2 μg/mL in *Mucor* spp. [1,40,53,54,55,56]. Also, in vivo activity for posaconazole have been reported with different efficacy among species [57]. Another triazole, isavuconazole, shows in vitro [58] and in vivo [59] activity against Mucorales, and its use is recommended as an alternative in those cases in which amphotericin B (a polyene compound that alters the membrane permeability) is inappropriate. Amphotericin B is an old antifungal compound with several toxic effects (kidney and liver); however, it is currently the most efficient antifungal compound against mucormycosis [57,60]. Regarding itraconazole, although it is not used for mucormycosis treatment, some investigations demonstrated the in vitro and in vivo activity of this drug, showing a species-specific efficacy in both conditions [53]. 

The mechanisms of antifungal resistance have been widely studied in organisms such as *Candida* and *Aspergillus*, and include mechanisms that affect either the target, by increasing its amount or decreasing azole inhibition, or the transport of the drug [61,62]. Mutations in the zinc cluster transcription factors (ZCFs), for instance, the Tac1 transcription factor, triggers overexpression of CDR1 and CDR2 genes, two efflux pumps that transport drugs extracellularly, limiting the interaction between triazoles and its target proteins [63,64]. Similarly, the Mrr1 transcription factor activates the expression of the Mdr1 transporter [65,66]. 

The lanosterol 14α-demethylase (CYP51 or Erg11), which is critical for ergosterol biosynthesis, is the target protein for triazole drugs and mutations found in isolates from patients are the cause of many triazole resistances. The first mutation reported was a point substitution (R467K) implicated in the heme group interaction with the triazole molecule. In the subsequent years, researchers found several other point mutations, although triazole affinity reduction was not uniform in all of them [67,68]. In the case of *Aspergillus*, a naturally occurring T301I substitution is the cause of its intrinsic resistance to fluconazole [69,70,71,72,73]. Combinations of different mutations are also involved in several mechanisms of antifungal resistances [74].

Interestingly, two paralogues of CYP51 are present in the genome of *M. circinelloides* (named CYP51 F1 and CYP51 F5), like it was reported in *Aspergillus fumigatus* [75]; and similarly, point mutations in these proteins might play a key role in the reduction of the affinity to one or more types of triazole drugs. In this sense, a recent study has shed some light on this issue [76]. The sequence analysis of both CYP51 F1 and CYP51 F5 revealed the presence of a naturally occurring point mutation in the CYP51 F5 gene that might explain the intrinsic resistance of Mucorales to fluconazole and voriconazole (short-tailed triazoles). This change, detected by multiple sequence alignment, consists of a phenylalanine substitution in position 129 of the protein sequence (Y129F). Although the specific functional role of the Y129F substitution in Mucorales requires further investigation, the structural analysis of this protein suggests a putative mechanism to explain short-tailed azole resistance [77]. Based on the resolved structure of the *Saccharomyces cerevisiae* lanosterol 14α-demethylase, it was possible to correlate Y129F with Y140F in the *S. cerevisiae* protein. In the yeast model, the substitution confers resistance to short-tailed triazoles because of the loss of a hydrogen bond among the hydroxyl of Y140, the tertiary alcohol short-tailed triazoles and a heme propionate (via a highly conserved water molecule), resulting in a decreased affinity of the lanosterol 14α-demethylase for triazole drugs. In contrast, the loss of this hydrogen bond has no impact on the binding of mid- and long-tailed triazole drugs, as they can fill the space occupied by the water molecule [77]. The validation of this mechanism in Mucorales will provide a new and promising opportunity to re-design our current triazole drugs, and make them more efficient against the CYP51 F5 proteins of Mucorales.

## 6. Omic Technologies to Find New Virulence Factors

Reverse genetics approaches have identified several virulence factors in Mucorales. The deletion in Mucorales of genes characterized previously in other organisms led to the identification of virulence-reduced phenotypes in mutants for genes involved in iron uptake [17,78], ADP-ribosylation factor (Arf) [79] and Calcineurin [29]. Nowadays, the availability of mucoralean genomes and the knowledge of RNAi mechanisms have allowed strategies of structural and functional genomics to identify a growing number of new virulence factors in these fungi. 

Comparative genomics approaches have shown its potential for discovering new virulence factors, including those whose function is unknown. A recent example is a comparison between the genomes of the *M. circinelloides* virulent strain CBS277.49 and the strain with reduced virulent NRRL3631. This genomic comparison allowed the identification of 543 absent genes and 230 discontiguous protein-coding sequences in the avirulent strain [80]. Attention was focused on extracellular proteins because of their strong association with pathogenic potential. One of these proteins with unknown function, encoded by the gene ID112092, resulted in an essential role in virulence because knock-out mutants in CBS277.49 for gene ID112092 exhibited a similar attenuated virulence in a murine model as NRRL3631, making evident the potential of this genomic approach [80]. Other studies analyzed the genomes of 30 mucormycosis-causing fungi, evidencing a correlation between the copy number of *cotH* genes (Inner spore coat Protein H, involved in the assembly of several proteins in the inner and outer layer of the spore coat, described in Section 8) and clinical prevalence [81]. 

Similarly, a genome mining and transcriptomic analysis in *L. corymbifera* identified new genes related to virulence, along with other factors previously described. Among them, the study revealed genes involved in iron-uptake, a large number of putative secreted proteases, and four families of transcription factors (TFs) not described before in fungal species [19]. Regarding the transcription factors, two of them (LCor02851.1 and LCor8197.1 of the WRKY DNA-binding family, described in plants and bacteria) were up-regulated under hypoxic conditions, whereas a third (LCor09690.1) was down-regulated [19]. These studies enlighten the relevance of TFs during infection. Host invasion requires complex regulation of gene expression to survive under the challenging conditions inside the host. In this sense, pathogenic Mucorales have a specific set of TFs driving the response to the host adverse conditions, becoming suitable target elements to develop specific antifungal drugs [13]. 

Functional genomic strategy based on the generation of RNAi high-throughput libraries using silencing plasmids and a host model like *Galleria mellonella* has allowed fast screenings and identification of virulence in *M. circinelloides*. The first whole-genome screening using this methodology led to the identification of two new virulence factors, the genes *mcplD* and *mcmyo5* [12]. Transformant carrying plasmids that triggered silencing of these genes showed reduced virulence in *G. mellonella*, which later was confirmed in a murine model infected with knockout mutants. The gene *mcplD* codes for a well-conserved Phospholipase D enzyme, which is involved in germination and hyphal growth of *M. circinelloides*, while the gene *mcmyo5* encodes an essential myosin transporter of Myosin V class transporters. Although the new virulence factors identified by this RNAi approach are highly conserved genes, the presence of a specific fungal cargo domain in *mcmyo5* could represent a potential specific antifungal target [12]. 

A general complication that affects all these omics technologies is the whole genome duplication at the early stages of the evolution of Mucorales. It was first described in *R. delemar* [82], and later in *M. circinelloides* and *Phycomyces blakesleeanus* [83]. Thus, many of the promising candidate genes identified with the omics technologies usually belong to gene families with several paralogues, representing an additional difficulty for further functional analysis [42,43,44]. Nevertheless, all these genomic studies have demonstrated their potential to identify and characterize new virulence factors in Mucorales, and many candidates are waiting for further studies to know in-depth their function in the infection.

## 7. Mucoralean Gene Response to Host Innate Immunity

Mucormycosis affects mainly immunocompromised individuals, especially those suffering from severe and prolonged neutropenia, hematological malignancies, poorly controlled diabetes, and other diseases requiring immunosuppressive treatments [84,85]. The predominant mode to develop the infection is through inhalation of fungal asexual spores, leading to the most prevalent forms of infection: rhino-orbito-cerebral and pulmonary infections [3]. However, the fungal spores must overcome the innate immune system in order to germinate and develop the angioinvasive form of infection. During this struggle, macrophages and neutrophils are recruited to the site of infection to protect their host. The phagocytes internalize the fungal spores, halting spore germination and hyphal growth in zebrafish, rabbit, and mouse models [86,87,88,89]. 

Preventing systemic infections depends strictly on the success of the early immune response, explaining why innate immune defects and immunosuppression are major predisposing factors in developing mucormycosis. Interestingly, the behavior and fate of the fungal spores during host-pathogen interaction are genus-specific. Macrophages from healthy mice inhibit germination in *R. oryzae*, as opposed to macrophages from immunosuppressed mice [88,90]. Despite this inhibition, spores from *Rhizopus* spp. survive phagocytosis and persist within the macrophages, arresting phagosome maturation by retaining melanin on their cell wall surface [91]. In contrast, spores from a virulent strain of *L. corymbifera* are more frequently phagocytosed than those of an attenuated strain [92], suggesting that they could exploit macrophage phagocytosis to disseminate the infection. Similarly, spores from virulent *Mucor* spp. can survive and germinate after mice macrophage phagocytosis, while avirulent strains remain dormant [12,37,80]. However, the molecular and genetic response of both host and pathogen during this interaction is not yet well defined and constitutes a promising field of research. 

Several studies have characterized the host-pathogen genetic reaction during phagocytosis. The first host-Mucorales gene expression analysis was conducted in human polymorphonuclear neutrophils. Upon *R. oryzae* infection, these phagocytes launch an early pro-inflammatory response that involves the induction of Toll-like receptor 2 (TLR2). The increasing advances in high-throughput RNA sequencing allowed the study of the host-Mucorales genetic interactions in mammalian models [93]. A large-scale transcriptomic survey was performed during the infection of human airway epithelial cells with *Rhizopus* and *Mucor* spp., mimicking the initial host-pathogen encounter after spore inhalation. This study revealed that both genera elicit a similar host response, a robust pro-inflammatory reaction. This response involves the platelet-derived growth factor receptor B (PDGFRB) signaling pathway in the core response to mucoralean infection that damages barrier host cells [81]. Subsequent transcriptomic studies confirmed the proinflammatory reaction to virulent mucoralean species. *Mucor*-infected zebrafish larvae [94] and mouse macrophages [13] elicit a robust pro-inflammatory response, but only against *M. circinelloides* virulent strain, whereas the avirulent strain failed to induce an inflammatory reaction. In both mouse and zebrafish macrophages, *M. circinelloides* virulent strains induced pro-apoptotic pathways, suggesting that inducing apoptosis could represent a key virulence mechanism in *Mucor* spp. In contrast, *Rhizopus* spp. induce an iron restriction response in human macrophages, and its melanin arrests phagosome maturation and inhibits apoptotic pathways by constitutively activation of Akt/PI3K signaling [91]. These differences could explain why spores from virulent *Mucor* spp. can germinate and kill healthy macrophages, while those from *Rhizopus* spp. remain dormant and persist inside the phagosome.

From the pathogen genetic response, each species seems to adapt differently to the host environment. The expression profile of opposite *M. circinelloides* pathotypes, virulent and avirulent strains, revealed that an ATF-regulated gene network is essential for the virulent strain to survive and germinate inside the phagosome [13]. In this response, the impoverished intraphagosomal environment requires significant metabolic changes, as well as cytoskeletal and cell surface remodeling during the germination process. It is proposed that *Mucor* spp. thrive within the macrophages, taking advantage of the acidic pH to induce germination and hyphal growth [13]. This mechanism could be responsible for the positive correlation between virulence and the rate of phagocytosis observed in *L. corymbifera*, explaining why the most virulent strains are those readily phagocytosed [92]. On the other hand, the genetic response of *Rhizopus* spp. to phagocytosis comprises several genes related to iron metabolism, especially those involved in the high-affinity iron uptake mechanism [91], which is in accordance with their dormant state. 

Although each specie in the lineage Mucorales show a distinct behavior in their interaction with phagocytic cells, there is some common ground in all species, either virulent or with attenuated virulence, like their genetic reaction to fight against nutritional immunity. Hence, improvements in the host defense mechanisms that are involved in nutritional immunity should guide the development of possible treatments against mucormycosis.

## 8. *cotH* Gene Family, A Distinctive Virulence Factor in Mucorales

CotH are atypical protein kinases that constitute a structural component of spores of different human pathogens, including prokaryotes and eukaryotes [95,96]. In *Bacillus subtilis*, CotH regulates the integrity of the spore, as CotH mutant spores showed a deficiency in germination [97]. CotH orthologs from *B. subtilis* were identified in basal fungi, showing a distribution located mainly on the surface of the spore coat [97]. Later, new studies found that these proteins are not present in the spore of noninvasive pathogens, whereas it is a common feature in pathogenic strains of Mucorales associated with mucormycosis [98]. In addition, the copy number of *cotH* genes correlates with the pathogenic potential of the different species isolated from patients suffering mucormycosis. Thus, the frequently isolated species from the genus *Rhizopus* have six to seven copies, the less frequently isolated genera harbor three to seven copies, and the rarely isolated species have only one or two copies [81]. 

The genus *Rhizopus* is the principal causative agent of mucormycosis, and *R. delemar* is the most frequently isolated species from infected patients. *R. delemar* has eight different CotH proteins, and six of them (CotH1-3 and CotH6-8) present the aminoacidic sequence “MGQTNDGAYRDPTDNN” exposed on the spore coat [81]. This motif is considered the main determinant factor for the specific interaction between the fungal spores and the host endothelial cells, being essential to drive the host invasion [99]. The expression of *cotH* genes in *R. delemar* during the interaction with hot cells confirms their contribution to the infection [3]. Other species from the genera *Mucor* and *Apophysomyces* that are frequently isolated from invasive infections also correlate with the presence of a high number of *cotH* genes in their genomes [100]. Out of the order Mucorales, human fungal pathogens like *Candida albicans* and *A. fumigatus* do not have homologs of CotH proteins. Instead, these kind of fungi use agglutinin-like proteins (Als) and thaumatin (CalA) to invade the host [101]. Thus, CotH protein kinases are specifically conserved in Mucorales, giving them the ability to adhere and cause damage in the host tissues [101].

The mechanism of tissue invasion mediated by CotH proteins relies on the interaction of CotH2-CotH3 with GRP78 (glucose-regulated protein 78) in the surface of the epithelial cells [98]. CotH3 has the highest affinity with GRP78, followed by CotH2. GRP78 is a heat-shock protein of the HSP70 family, usually located in the endoplasmic reticulum (ER) of the endothelial cells [102]. However, stress conditions induce overexpression of GRP78 and its release from the ER to the cell surface, where it can interact with the proteins CotH2 and CotH3 [102]. GRP78 was identified as a protein that binds to mucoralean germlings during the invasion, but not to the spores [103]. Studies of heterologous expression of CotH2 and CotH3 in *S. cerevisiae*, along with the generation of *R. delemar* strains with reduced expression of these two proteins, evidenced their role in the process of adhesion, although adhesion is not required for their role in tissue invasion [98]. The importance of both CotH and GRP78 in the development of mucormycosis was also studied using *in vivo* models. In mice with DKA, the presence of high concentrations of iron and glucose produced GRP78 overexpression and higher susceptibility to the disease [103]. Correlatively, reduced expression of CotH2 and CotH3 decreases virulence of *R. delemar*, even when the expression of GRP78 is over the normal levels [98]. 

The therapeutic potential of CotH protein kinases relies on both their essential role during the infection and their specificity in the order Mucorales [104]. Thus, a therapy based on monoclonal antibodies showed positive results in mice. Treatments with these anti-CotH3 antibodies and the conventional antifungal drugs achieved 100% survival in mice against many mucoralean species [105]. Moreover, *cotH* genes can serve as a diagnostic target using PCR technology, which is more reliable than the classic methods and allows detection in the early stages of mucormycosis [106].

## 9. Concluding Remarks and Future Perspectives

Genetics in fungi of the order Mucorales has a history of high initial expectations, the subsequent disillusionment, and a further re-emergence. It started sponsored by Max Delbrück, a Nobel-Laureated for his studies concerning the replication and the genetic structure of viruses. After the Nobel award in 1969, Max Delbrück radically changed his research line and adopted *P. blakesleeanus* as a new study model, describing this fungus as the simplest eukaryote to investigate complex sensory systems. For 25 years, he made significant advances and created a school of enthusiastic successors. However, the reluctance of this organism to accept the genetic transformation always hampered them. In 1984, the *M. circinelloides* was found as a unique exception in the group that could be genetically transformed [107]. Nevertheless, by that time, most of the investigators studying fungal biology had established their research using different models such as *Saccharomyces*, *Aspergillus*, and *Candida*, and only a few groups remained to study the order Mucorales. The renewed interest in these fungi started by the last years of the first decade of this century when the medical community alerted on the emerging mucormycosis. 

In the last ten years, many studies are unveiling the genes behind the virulence of Mucorales, showing perspectives as a future hot-topic field, and promising the development of new treatments against mucormycosis. Among these studies, several of them focused their research on the iron uptake systems. Iron is essential for most organisms, and its role as a virulence factor was known in many pathogens before Mucorales. Despite this previous knowledge, there are no well-established therapies based on iron-chelators, although some clinical cases describe the use of deferasirox (an iron-chelating agent) as salvage therapy [108]. The new studies described here identified a link between the iron uptake system and dimorphism, indicating the presence of regulatory factors controlling both processes. These still unknown factors could represent targets with higher perspectives for new treatments, as they would compromise two essential processes involved in virulence. The thorough studies on dimorphism and gene silencing fructified with the unveiling of a new mechanism of antifungal resistance based on the epigenetic silencing of target genes. The antifungal compound used in these studies was the FK-506 (Tacrolimus), a compound blocking the pathogen in the avirulent yeast morphology. Unfortunately, this compound presents a strong immunosuppressive effect on patients, preventing its use as an antifungal therapy [109]. Nevertheless, new compounds could be designed for the same or other targets in the calcineurin pathway, and in combination with gene silencing inhibitors, it might represent a promising new therapy. Another rising hypothesis proposes the use of specific antibodies against conserved regions of the CotH proteins, and although its effectiveness has been tested only in animal models, the current results anticipate an optimistic output. 

Most of these studies are focusing their effort on target virulence factors described in other pathogens before Mucorales, which is a straightforward strategy. However, the unusual virulence of Mucorales and their outstanding antifungal resistance to different types of compounds suggest a multifactorial combination of particularities as an intrinsic feature in the virulence of this group of fungi. In this sense, the first whole-genome sequencing of the species *P. blakesleeanus*, *R. delemar*, and *M. circinelloides* revealed a noteworthy number of “unknown function genes” [83]. Undoubtedly, this unknown and non-conserved part of the genomes of Mucorales must play a critical role in shaping the unique behavior of these fungi, and therefore, it becomes the ideal target to design new therapies against mucormycosis. Several studies cited above focused their work on comparative genomic analyses, transcriptomic profiling, and genome response during host-pathogen interactions. These studies contributed with extensive lists of candidates related to virulence, including many with unknown function. However, most of these studies concentrated their efforts on conserved genes with predicted functions because it facilitates further investigations to unveil their role in virulence. A resolute attempt to investigate specific genes of Mucorales must be conducted in the next years that will likely uncover new and efficient strategies to combat mucormycosis.

## References

[1] Dannaoui E. (2017). Antifungal resistance in mucorales. Int. J. Antimicrob. Agents.

[2] Petrikkos G., Skiada A., Lortholary O., Roilides E., Walsh T.J., Kontoyiannis D.P. (2012). Epidemiology and Clinical Manifestations of Mucormycosis. Clin. Infect. Dis..

[3] Prakash H., Chakrabarti A. (2019). Global epidemiology of mucormycosis. J. Fungi.

[4] Chayakulkeeree M., Ghannoum M.A., Perfect J.R. (2006). Zygomycosis: The re-emerging fungal infection. Eur. J. Clin. Microbiol. Infect. Dis..

[5] Sridhara S.R., Paragache G., Panda N.K., Chakrabarti A. (2005). Mucormycosis in immunocompetent individuals: An increasing trend. J. Otolaryngol..

[6] Gutiérrez A., López-García S., Garre V. (2011). High reliability transformation of the basal fungus Mucor circinelloides by electroporation. J. Microbiol. Methods.

[7] Xu S., Zhou Z., Du G., Zhou J., Chen J. (2014). Efficient transformation of *Rhizopus delemar* by electroporation of germinated spores. J. Microbiol. Methods.

[8] Baldin C., Ibrahim A.S. (2017). Molecular mechanisms of mucormycosis—The bitter and the sweet. PLoS Pathog..

[9] Hassan M.I.A., Voigt K. (2019). Pathogenicity patterns of mucormycosis: Epidemiology, interaction with immune cells and virulence factors. Med. Mycol..

[10] Ruiz-Vázquez R.M., Nicolás F.E., Torres-Martínez S., Garre V. (2015). Distinct RNAi Pathways in the Regulation of Physiology and Development in the Fungus Mucor circinelloides. Adv. Genet..

[11] Calo S., Shertz-Wall C., Lee S.C., Bastidas R.J., Nicolás F.E., Granek J.A., Mieczkowski P., Torres-Martínez S., Ruiz-Vázquez R.M., Cardenas M.E. (2014). Antifungal drug resistance evoked via RNAi-dependent epimutations. Nature.

[12] Trieu T.A., Navarro-Mendoza M.I., Pérez-Arques C., Sanchis M., Capilla J., Navarro-Rodriguez P., Lopez-Fernandez L., Torres-Martínez S., Garre V., Ruiz-Vázquez R.M. (2017). RNAi-Based Functional Genomics Identifies New Virulence Determinants in Mucormycosis. PLoS Pathog..

[13] Pérez-Arques C., Navarro-Mendoza M.I., Murcia L., Lax C., Martínez-García P., Heitman J., Nicolás F.E., Garre V. (2019). *Mucor circinelloides* thrives inside the phagosome through an Atf-mediated germination pathway. MBio.

[14] Bullen J.J. (2006). Natural resistance, iron and infection: A challenge for clinical medicine. J. Med. Microbiol..

[15] Gebremariam T., Lin L., Liu M., Kontoyiannis D.P., French S., Edwards J.E., Filler S.G., Ibrahim A.S. (2016). Bicarbonate correction of ketoacidosis alters host-pathogen interactions and alleviates mucormycosis. J. Clin. Investig..

[16] Carroll C.S., Grieve C.L., Murugathasan I., Bennet A.J., Czekster C.M., Liu H., Naismith J., Moore M.M. (2017). The rhizoferrin biosynthetic gene in the fungal pathogen *Rhizopus delemar* is a novel member of the NIS gene family. Int. J. Biochem. Cell Biol..

[17] Navarro-Mendoza M.I., Pérez-Arques C., Murcia L., Martínez-García P., Lax C., Sanchis M., Capilla J., Nicolás F.E., Garre V. (2018). Components of a new gene family of ferroxidases involved in virulence are functionally specialized in fungal dimorphism. Sci. Rep..

[18] Ibrahim A.S., Gebremariam T., Lin L., Luo G., Husseiny M.I., Skory C.D., Fu Y., French S.W., Edwards J.E., Spellberg B. (2010). The high affinity iron permease is a key virulence factor required for *Rhizopus oryzae* pathogenesis. Mol. Microbiol..

[19] Schwartze V.U., Winter S., Shelest E., Marcet-Houben M., Horn F., Wehner S., Linde J., Valiante V., Sammeth M., Riege K. (2014). Gene Expansion Shapes Genome Architecture in the Human Pathogen Lichtheimia corymbifera: An Evolutionary Genomics Analysis in the Ancient Terrestrial Mucorales (Mucoromycotina). PLoS Genet..

[20] Shirazi F., Kontoyiannis D.P., Ibrahim A.S. (2015). Iron starvation induces apoptosis in *Rhizopus oryzae* in vitro. Virulence.

[21] Haas H. (2003). Molecular genetics of fungal siderophore biosynthesis and uptake: The role of siderophores in iron uptake and storage. Appl. Microbiol. Biotechnol..

[22] Thieken A., Winkelmann G. (1992). Rhizoferrin: A complexone type siderophore of the Mucorales and entomophthorales (Zygomycetes). FEMS Microbiol. Lett..

[23] Liu M., Lin L., Gebremariam T., Luo G., Skory C.D., French S.W., Chou T.F., Edwards J.E., Ibrahim A.S. (2015). Fob1 and Fob2 Proteins Are Virulence Determinants of *Rhizopus oryzae* via Facilitating Iron Uptake from Ferrioxamine. PLoS Pathog..

[24] Ibrahim A.S., Gebermariam T., Fu Y., Lin L., Husseiny M.I., French S.W., Schwartz J., Skory C.D., Edwards J.E., Spellberg B.J. (2007). The iron chelator deferasirox protects mice from mucormycosis through iron starvation. J. Clin. Investig..

[25] Schwarz P., Cornely O.A., Dannaoui E. (2019). Antifungal combinations in Mucorales: A microbiological perspective. Mycoses.

[26] Kousser C., Clark C., Sherrington S., Voelz K., Hall R.A. (2019). *Pseudomonas aeruginosa* inhibits *Rhizopus microsporus* germination through sequestration of free environmental iron. Sci. Rep..

[27] Orlowski M. (1991). Mucor dimorphism. Microbiol. Rev..

[28] Wolff A.M., Appel K.F., Petersen J.B., Poulsen U., Arnau J. (2002). Identification and analysis of genes involved in the control of dimorphism in Mucor circinelloides (syn. racemosus). FEMS Yeast Res..

[29] Lee S.C., Li A., Calo S., Heitman J. (2013). Calcineurin plays key roles in the dimorphic transition and virulence of the human pathogenic zygomycete *Mucor circinelloides*. PLoS Pathog..

[30] Ruiz-Herrera J. (1994). Polyamines, DNA methylation, and fungal differentiation. Crit. Rev. Microbiol..

[31] Binder U., Navarro-Mendoza M.I., Naschberger V., Bauer I., Nicolas F.E., Pallua J.D., Lass-Flörl C., Garre V. (2018). Generation of a mucor circinelloides reporter strain—A promising new tool to study antifungal drug efficacy and mucormycosis. Genes.

[32] Navarro-Mendoza M.I., Pérez-Arques C., Panchal S., Nicolás F.E., Mondo S.J., Ganguly P., Pangilinan J., Grigoriev I.V., Heitman J., Sanyal K. (2019). Early Diverging Fungus Mucor circinelloides Lacks Centromeric Histone CENP-A and Displays a Mosaic of Point and Regional Centromeres. Curr. Biol..

[33] Nicolás F., Ruiz-Vázquez R. (2013). Functional Diversity of RNAi-Associated sRNAs in Fungi. Int. J. Mol. Sci..

[34] Nicolás F.E., Navarro-Mendoza M.I., Pérez-Arques C., López-García S., Navarro E., Torres-Martínez S., Garre V. (2018). Molecular tools for carotenogenesis analysis in the mucoral Mucor circinelloides. Methods in Molecular Biology.

[35] Lee S.C., Li A., Calo S., Inoue M., Tonthat N.K., Bain J.M., Louw J., Shinohara M.L., Erwig L.P., Schumacher M.A. (2015). Calcineurin orchestrates dimorphic transitions, antifungal drug responses and host-pathogen interactions of the pathogenic mucoralean fungus Mucor circinelloides. Mol. Microbiol..

[36] Boyce K.J., Andrianopoulos A. (2015). Fungal dimorphism: The switch from hyphae to yeast is a specialized morphogenetic adaptation allowing colonization of a host. FEMS Microbiol. Rev..

[37] Li C.H., Cervantes M., Springer D.J., Boekhout T., Ruiz-Vazquez R.M., Torres-Martinez S.R., Heitman J., Lee S.C. (2011). Sporangiospore size dimorphism is linked to virulence of *Mucor circinelloides*. PLoS Pathog..

[38] Ocampo J., Nuñez L.F., Silva F., Pereyra E., Moreno S., Garre V., Rossi S. (2009). A subunit of protein kinase a regulates growth and differentiation in the fungus *Mucor circinelloides*. Eukaryot Cell.

[39] Patiño-Medina J.A., Reyes-Mares N.Y., Valle-Maldonado M.I., Jácome-Galarza I.E., Pérez-Arques C., Nuñez-Anita R.E., Campos-García J., Anaya-Martínez V., Ortiz-Alvarado R., Ramírez-Díaz M.I. (2019). Heterotrimeric G-alpha subunits Gpa11 and Gpa12 define a transduction pathway that control spore size and virulence in *Mucor circinelloides*. PLoS ONE.

[40] Valle-Maldonado M.I., Jácome-Galarza I.E., Díaz-Pérez A.L., Martínez-Cadena G., Campos-García J., Ramírez-Díaz M.I., Reyes-De la Cruz H., Riveros-Rosas H., Díaz-Pérez C., Meza-Carmen V. (2015). Phylogenetic analysis of fungal heterotrimeric G protein-encoding genes and their expression during dimorphism in *Mucor circinelloides*. Fungal Biol..

[41] Chang Z., Billmyre R.B., Lee S.C., Heitman J. (2019). Broad antifungal resistance mediated by RNAi-dependent epimutation in the basal human fungal pathogen *Mucor circinelloides*. PLoS Genet..

[42] Calo S., Nicolas F.E., Vila A., Torres-Martinez S., Ruiz-Vazquez R.M. (2012). Two distinct RNA-dependent RNA polymerases are required for initiation and amplification of RNA silencing in the basal fungus *Mucor circinelloides*. Mol. Microbiol..

[43] De Haro J.P., Calo S., Cervantes M., Nicolás F.E., Torres-Martínez S., Ruiz-Vázquez R.M. (2009). A Single *dicer* Gene Is Required for Efficient Gene Silencing Associated with Two Classes of Small Antisense RNAs in *Mucor circinelloides*. Eukaryot Cell.

[44] Cervantes M., Vila A., Nicolás F.E., Moxon S., de Haro J.P., Dalmay T., Torres-Martínez S., Ruiz-Vázquez R.M. (2013). A Single Argonaute Gene Participates in Exogenous and Endogenous RNAi and Controls Cellular Functions in the Basal Fungus *Mucor circinelloides*. PLoS ONE.

[45] Nicolas F.E., Moxon S., de Haro J.P., Calo S., Grigoriev I.V., Torres-Martinez S., Moulton V., Ruiz-Vazquez R.M., Dalmay T. (2010). Endogenous short RNAs generated by Dicer 2 and RNA-dependent RNA polymerase 1 regulate mRNAs in the basal fungus *Mucor circinelloides*. Nucleic Acids Res..

[46] Nicolás F.E., Vila A., Moxon S., Cascales M.D., Torres-Martínez S., Ruiz-Vázquez R.M., Garre V. (2015). The RNAi machinery controls distinct responses to environmental signals in the basal fungus *Mucor circinelloides*. BMC Genom..

[47] Calo S., Nicolás F.E., Lee S.C., Vila A., Cervantes M., Torres-Martinez S., Ruiz-Vazquez R.M., Cardenas M.E., Heitman J. (2017). A non-canonical RNA degradation pathway suppresses RNAi-dependent epimutations in the human fungal pathogen *Mucor circinelloides*. PLoS Genet..

[48] Trieu T.A., Calo S., Nicolás F.E., Vila A., Moxon S., Dalmay T., Torres-Martínez S., Garre V., Ruiz-Vázquez R.M. (2015). A Non-canonical RNA Silencing Pathway Promotes mRNA Degradation in Basal Fungi. PLoS Genet..

[49] Chang Z., Heitman J. (2019). Drug-resistant epimutants exhibit organ-specific stability and induction during murine infections caused by the human fungal pathogen *Mucor circinelloides*. MBio.

[50] Groll A.H., Gea-Banacloche J.C., Glasmacher A., Just-Nuebling G., Maschmeyer G., Walsh T.J. (2003). Clinical pharmacology of antifungal compounds. Infect. Dis. Clin. N. Am..

[51] Lass-Flörl C. (2011). Triazole antifungal agents in invasive fungal infections: A comparative review. Drugs.

[52] Watson P.F., Rose M.E., Ellis S.W., England H., Kelly S.L. (1989). Defective sterol C5-6 desaturation and azole resistance: A new hypothesis for the mode of action of azole antifungals. Biochem. Biophys. Res. Commun..

[53] Vitale R.G., De Hoog G.S., Schwarz P., Dannaoui E., Deng S., Machouart M., Voigt K., Van De Sande W.W.J., Dolatabadi S., Meis J.F. (2012). Antifungal susceptibility and phylogeny of opportunistic members of the order Mucorales. J. Clin. Microbiol..

[54] Espinel-Ingroff A., Chakrabarti A., Chowdhary A., Cordoba S., Dannaoui E., Dufresne P., Fothergill A., Ghannoum M., Gonzalez G.M., Guarro J. (2015). Multicenter evaluation of MIC distributions for epidemiologic cutoff value definition to detect amphotericin B, posaconazole, and itraconazole resistance among the most clinically relevant species of Mucorales. Antimicrob. Agents Chemother..

[55] Maurer E., Binder U., Sparber M., Lackner M., Caramalho R., Lass-Flörl C. (2015). Susceptibility profiles of amphotericin B and posaconazole against clinically relevant mucorales species under hypoxic conditions. Antimicrob. Agents Chemother..

[56] Chowdhary A., Singh P.K., Kathuria S., Hagen F., Meis J.F. (2015). Comparison of the EUCAST and CLSI broth microdilution methods for testing isavuconazole, posaconazole, and amphotericin b against molecularly identified Mucorales species. Antimicrob. Agents Chemother..

[57] Luo G., Gebremariam T., Lee H., French S.W., Wiederhold N.P., Patterson T.F., Filler S.G., Ibrahim A.S. (2013). Efficacy of liposomal amphotericin B and posaconazole in intratracheal models of murine mucormycosis. Antimicrob. Agents Chemother..

[58] Arendrup M.C., Jensen R.H., Meletiadis J. (2015). In vitro activity of isavuconazole and comparators against clinical isolates of the Mucorales order. Antimicrob. Agents Chemother..

[59] Luo G., Gebremariam T., Lee H., Edwards J.E., Kovanda L., Ibrahim A.S. (2014). Isavuconazole therapy protects immunosuppressed mice from mucormycosis. Antimicrob. Agents Chemother..

[60] Amphotericin B nephrotoxicity|Journal of Antimicrobial Chemotherapy|Oxford Academic. https://academic.oup.com/jac/article/49/suppl_1/37/2473434.

[61] Nishimoto A.T., Sharma C., Rogers P.D. (2019). Molecular and genetic basis of azole antifungal resistance in the opportunistic pathogenic fungus *Candida albicans*. J. Antimicrob. Chemother..

[62] Chowdhary A., Sharma C., Hagen F., Meis J.F. (2014). Exploring azole antifungal drug resistance in *Aspergillus fumigatus* with special reference to resistance mechanisms. Future Microbiol..

[63] Prasad R., De Wergifosse P., Goffeau A., Balzi E. (1995). Molecular cloning and characterization of a novel gene of *Candida albicans*, CDR1, conferring multiple resistance to drugs and antifungals. Curr. Genet..

[64] Sanglard D., Kuchler K., Ischer F., Pagani J.L., Monod M., Bille J. (1995). Mechanisms of resistance to azole antifungal agents in *Candida albicans* isolates from AIDS patients involve specific multidrug transporters. Antimicrob. Agents Chemother..

[65] Franz R., Kelly S.L., Lamb D.C., Kelly D.E., Ruhnke M., Morschhäuser J. (1998). Multiple molecular mechanisms contribute to a stepwise development of fluconazole resistance in clinical *Candida albicans* strains. Antimicrob. Agents Chemother..

[66] Dunkel N., Blass J., Rogers P.D., Morschhäuser J. (2008). Mutations in the multi-drug resistance regulator MRR1, followed by loss of heterozygosity, are the main cause of MDR1 overexpression in fluconazole-resistant *Candida albicans* strains. Mol. Microbiol..

[67] White T.C. (1997). The presence of an R467K amino acid substitution and loss of allelic variation correlate with an azole-resistant lanosterol 14alpha demethylase in *Candida albicans*. Antimicrob. Agents Chemother..

[68] Sanglard D., Ischer F., Koymans L., Bille J. (1998). Amino acid substitutions in the cytochrome P-450 lanosterol 14α-demethylase (CYP51A1) from azole-resistant *Candida albicans* clinical isolates contribute to resistance to azole antifungal agents. Antimicrob. Agents Chemother..

[69] Leonardelli F., Macedo D., Dudiuk C., Cabeza M.S., Gamarra S., Garcia-Effron G. (2016). *Aspergillus fumigatus* Intrinsic Fluconazole Resistance Is Due to the Naturally Occurring T301I Substitution in Cyp51Ap. Antimicrob. Agents Chemother..

[70] Snelders E., Karawajczyk A., Schaftenaar G., Verweij P.E., Melchers W.J.G. (2010). Azole resistance profile of amino acid changes in *Aspergillus fumigatus* CYP51A based on protein homology modeling. Antimicrob. Agents Chemother..

[71] Diaz-Guerra T.M., Mellado E., Cuenca-Estrella M., Rodriguez-Tudela J.L. (2003). A point mutation in the 14α-sterol demethylase gene cyp51a contributes to itraconazole resistance in *Aspergillus fumigatus*. Antimicrob. Agents Chemother..

[72] Abdolrasouli A., Rhodes J., Beale M.A., Hagen F., Rogers T.R., Chowdhary A., Meis J.F., Armstrong-James D., Fisher M.C. (2015). Genomic context of azole resistance mutations in *Aspergillus fumigatus* determined using whole-genome sequencing. MBio.

[73] Hagiwara D., Watanabe A., Kamei K., Goldman G.H. (2016). Epidemiological and Genomic Landscape of Azole Resistance Mechanisms in Aspergillus Fungi. Front. Microbiol..

[74] Warrilow A.G., Nishimoto A.T., Parker J.E., Price C.L., Flowers S.A., Kelly D.E., David Rogers P., Kelly S.L. (2019). The evolution of Azole resistance in *Candida albicans* Sterol 14-demethylase (CYP51) through incremental amino acid substitutions. Antimicrob. Agents Chemother..

[75] Mellado E., Diaz-Guerra T.M., Cuenca-Estrella M., Rodriguez-Tudela J.L. (2001). Identification of two different 14-alpha sterol demethylase-related genes (cyp51A and cyp51B) in *Aspergillus fumigatus* and other Aspergillus species. J. Clin. Microbiol..

[76] Caramalho R., Tyndall J.D.A., Monk B.C., Larentis T., Lass-Flörl C., Lackner M. (2017). Intrinsic short-tailed azole resistance in mucormycetes is due to an evolutionary conserved aminoacid substitution of the lanosterol 14α-demethylase. Sci. Rep..

[77] Sagatova A.A., Keniya M.V., Wilson R.K., Sabherwal M., Tyndall J.D.A., Monk B.C. (2016). Triazole resistance mediated by mutations of a conserved active site tyrosine in fungal lanosterol 14α-demethylase. Sci. Rep..

[78] Fu Y., Lee H., Collins M., Tsai H.F., Spellberg B., Edwards J.E., Kwon-Chung K.J., Ibrahim A.S. (2004). Cloning and functional characterization of the *Rhizopus oryzae* high affinity iron permease (*rFTR1*) gene. FEMS Microbiol. Lett..

[79] Patiño-Medina J.A., Maldonado-Herrera G., Pérez-Arques C., Alejandre-Castañeda V., Reyes-Mares N.Y., Valle-Maldonado M.I., Campos-García J., Ortiz-Alvarado R., Jácome-Galarza I.E., Ramírez-Díaz M.I. (2018). Control of morphology and virulence by ADP-ribosylation factors (Arf) in *Mucor circinelloides*. Curr. Genet..

[80] López-Fernández L., Sanchis M., Navarro-Rodríguez P., Nicolás F.E., Silva-Franco F., Guarro J., Garre V., Navarro-Mendoza M.I., Pérez-Arques C., Capilla J. (2018). Understanding *Mucor circinelloides* pathogenesis by comparative genomics and phenotypical studies. Virulence.

[81] Chibucos M.C., Soliman S., Gebremariam T., Lee H., Daugherty S., Orvis J., Shetty A.C., Crabtree J., Hazen T.H., Etienne K.A. (2016). An integrated genomic and transcriptomic survey of mucormycosis-causing fungi. Nat. Commun..

[82] Ma L.-J., Ibrahim A.S., Skory C., Grabherr M.G., Burger G., Butler M., Elias M., Idnurm A., Lang B.F., Sone T. (2009). Genomic Analysis of the Basal Lineage Fungus *Rhizopus oryzae* Reveals a Whole-Genome Duplication. PLoS Genet..

[83] Corrochano L.M., Kuo A., Marcet-Houben M., Polaino S., Salamov A., Villalobos-Escobedo J.M., Grimwood J., Álvarez M.I., Avalos J., Bauer D. (2016). Expansion of Signal Transduction Pathways in Fungi by Extensive Genome Duplication. Curr. Biol..

[84] Roden M.M., Zaoutis T.E., Buchanan W.L., Knudsen T.A., Sarkisova T.A., Schaufele R.L., Sein M., Sein T., Chiou C.C., Chu J.H. (2005). Epidemiology and Outcome of Zygomycosis: A Review of 929 Reported Cases. Clin. Infect. Dis..

[85] Ibrahim A.S., Spellberg B., Walsh T.J., Kontoyiannis D.P. (2012). Pathogenesis of mucormycosis. Clin. Infect. Dis..

[86] Inglesfield S., Jasiulewicz A., Hopwood M., Tyrrell J., Youlden G., Mazon-Moya M., Millington O.R., Mostowy S., Jabbari S., Voelz K. (2018). Robust phagocyte recruitment controls the opportunistic fungal pathogen *Mucor circinelloides* in innate granulomas In Vivo. MBio.

[87] Sheldom W.H., Bauer H. (1959). The development of the acute inflammatory response to experimental cutaneous mucormycosis in normal and diabetic rabbits. J. Exp. Med..

[88] Waldorf A.R., Ruderman N., Diamond R.D. (1984). Specific susceptibility to mucormycosis in murine diabetes and bronchoalveolar macrophage defense against *Rhizopus*. J. Clin. Investig..

[89] Voelz K., Gratacap R.L., Wheeler R.T. (2015). A zebrafish larval model reveals early tissue-specific innate immune responses to *Mucor circinelloides*. Dis. Model. Mech..

[90] Waldorf A.R., Levitz S.M., Diamond R.D. (1984). In vivo bronchoalveolar macrophage defense against *Rhizopus oryzae* and *Aspergillus fumigatus*. J. Infect. Dis..

[91] Andrianaki A.M., Kyrmizi I., Thanopoulou K., Baldin C., Drakos E., Soliman S.S.M., Shetty A.C., McCracken C., Akoumianaki T., Stylianou K. (2018). Iron restriction inside macrophages regulates pulmonary host defense against *Rhizopus* species. Nat. Commun..

[92] Kraibooj K., Park H.-R., Dahse H.-M., Skerka C., Voigt K., Figge M.T. (2014). Virulent strain of *Lichtheimia corymbifera* shows increased phagocytosis by macrophages as revealed by automated microscopy image analysis. Mycoses.

[93] Westermann A.J., Barquist L., Vogel J. (2017). Resolving host–pathogen interactions by dual RNA-seq. PLoS Pathog..

[94] López-Muñoz A., Nicolás F.E., García-Moreno D., Pérez-Oliva A.B., Navarro-Mendoza M.I., Hernández-Oñate M.A., Herrera-Estrella A., Torres-Martínez S., Ruiz-Vázquez R.M., Garre V. (2018). An Adult Zebrafish Model Reveals that Mucormycosis Induces Apoptosis of Infected Macrophages. Sci. Rep..

[95] McKenney P.T., Driks A., Eichenberger P. (2013). The Bacillus subtilis endospore: Assembly and functions of the multilayered coat. Nat. Rev. Microbiol..

[96] Nguyen K.B., Sreelatha A., Durrant E.S., Lopez-Garrido J., Muszewska A., Dudkiewicz M., Grynberg M., Yee S., Pogliano K., Tomchick D.R. (2016). Phosphorylation of spore coat proteins by a family of atypical protein kinases. Proc. Natl. Acad. Sci. USA.

[97] Saggese A., Scamardella V., Sirec T., Cangiano G., Isticato R., Pane F., Amoresano A., Ricca E., Baccigalupi L. (2014). Antagonistic role of CotG and CotH on spore germination and coat formation in Bacillus subtilis. PLoS ONE.

[98] Gebremariam T., Liu M., Luo G., Bruno V., Phan Q.T., Waring A.J., Edwards J.E., Filler S.G., Yeaman M.R., Ibrahim A.S. (2014). CotH3 mediates fungal invasion of host cells during mucormycosis. J. Clin. Investig..

[99] Lebreton A., Meslet-Cladière L., Morin-Sardin S., Coton E., Jany J.L., Barbier G., Corre E. (2019). Comparative analysis of five *Mucor* species transcriptomes. Genomics.

[100] Challa S. (2019). Mucormycosis: Pathogenesis and Pathology. Curr. Fungal Infect. Rep..

[101] Liu H., Lee M.J., Solis N.V., Phan Q.T., Swidergall M., Ralph B., Ibrahim A.S., Sheppard D.C., Filler S.G. (2016). *Aspergillus fumigatus* CalA binds to integrin α5β1 and mediates host cell invasion. Nat. Microbiol..

[102] Ibrahim I.M., Abdelmalek D.H., Elfiky A.A. (2019). GRP78: A cell’s response to stress. Life Sci..

[103] Liu M., Spellberg B., Phan Q.T., Fu Y., Fu Y., Lee A.S., Edwards J.E., Filler S.G., Ibrahim A.S. (2010). The endothelial cell receptor GRP78 is required for mucormycosis pathogenesis in diabetic mice. J. Clin. Investig..

[104] Alspaugh J.A. (2014). Hostile takeover: Fungal protein promotes host cell invasion. J. Clin. Investig..

[105] Gebremariam T., Alkhazraji S., Soliman S.S.M., Gu Y., Jeon H.H., Zhang L., French S.W., Stevens D.A., Edwards J.E., Filler S.G. (2019). Anti-CotH3 antibodies protect mice from mucormycosis by prevention of invasion and augmenting opsonophagocytosis. Sci. Adv..

[106] Baldin C., Soliman S.S.M., Jeon H.H., Alkhazraji S., Gebremariam T., Gu Y., Bruno V.M., Cornely O.A., Leather H.L., Sugrue M.W. (2018). PCR-based approach targeting mucorales-specific gene family for diagnosis of mucormycosis. J. Clin. Microbiol..

[107] Van Heeswijck R., Roncero M.I.G. (1984). High frequency transformation of *Mucor* with recombinant plasmid DNA. Carlsberg Res. Commun..

[108] Reed C., Ibrahim A., Edwards J.E., Walot I., Spellberg B. (2006). Deferasirox, an iron-chelating agent, as salvage therapy for rhinocerebral mucormycosis. Antimicrob. Agents Chemother..

[109] Hooks M.A. (1994). Tacrolimus, a new immunosuppressant—A review of the literature. Ann. Pharmacother..

